# Overcoming Resistance to Platinum-Based Drugs in Ovarian Cancer by Salinomycin and Its Derivatives—An In Vitro Study

**DOI:** 10.3390/molecules25030537

**Published:** 2020-01-26

**Authors:** Marcin Michalak, Michał Stefan Lach, Michał Antoszczak, Adam Huczyński, Wiktoria Maria Suchorska

**Affiliations:** 1 Department of Radiation Therapy and Gynecologic Oncology, Greater Poland Cancer Centre, Garbary 15, 61–866 Poznań, Poland; marcin.michalak@wco.pl; 2 Radiobiology Lab, Greater Poland Cancer Centre, Garbary 15, 61–866 Poznań, Poland; 3 The Postgraduate School of Molecular Medicine, Medical University of Warsaw, Księcia Trojdena 2a, 02–109 Warsaw, Poland; 4 Department of Electroradiology, Poznań University of Medical Sciences, Garbary 15, 61–866 Poznań, Poland; 5 Department of Medical Chemistry, Faculty of Chemistry, Adam Mickiewicz University, Uniwersytetu Poznańskiego 8, 61–614 Poznań, Poland; michant@amu.edu.pl (M.A.); adhucz@amu.edu.pl (A.H.)

**Keywords:** salinomycin, anticancer activity, overcoming drug resistance, tumor specificity, synergy, 5-fluorouracil, gemcitabine, amides/esters, ovarian cancer

## Abstract

Polyether ionophore salinomycin (SAL) and its semi-synthetic derivatives are recognized as very promising anticancer drug candidates due to their activity against various types of cancer cells, including multidrug-resistant populations. Ovarian cancer is the deadliest among gynecologic malignancies, which is connected with the development of chemoresistant forms of the disease in over 70% of patients after initial treatment regimen. Thus, we decided to examine the anticancer properties of SAL and selected SAL derivatives against a series of drug-sensitive (A2780, SK-OV-3) and derived drug-resistant (A2780 CDDP, SK-OV-3 CDDP) ovarian cancer cell lines. Although SAL analogs showed less promising IC_50_ values than SAL, they were identified as the antitumor agents that significantly overcome the resistance to platinum-based drugs in ovarian cancer, more potent than unmodified SAL and commonly used anticancer drugs—5-fluorouracil, gemcitabine, and cisplatin. Moreover, when compared with SAL used alone, our experiments proved for the first time increased selectivity of SAL-based dual therapy with 5-fluorouracil or gemcitabine, especially towards A2780 cell line. Looking closer at the results, SAL acted synergistically with 5-fluorouracil towards the drug-resistant A2780 cell line. Our results suggest that combinations of SAL with other antineoplastics may become a new therapeutic option for patients with ovarian cancer.

## 1. Introduction

Despite developing new therapeutic strategies and extensive knowledge about tumor biology, ovarian cancer (OvCa) remains a leading cause of mortality among gynecologic malignancies; asymptomatic early stages and lack of ambiguous biomarkers enabling detection of the disease lead to late diagnosis, mostly at stage III and IV [1,2]. Five-year overall survival for advanced OvCa is approximately 30% [3]. Most of the patients respond well to radical surgery and either neoadjuvant or adjuvant chemotherapy. However, 75% of patients develop recurrence [4]. Prognosis for patients with the recurrent disease is poor, especially for those diagnosed with the recurrence earlier than six months after completion of the initial platinum-based therapy [5]. This state is called platinum-resistance in opposition to platinum-sensitive patients who develop recurrence more than six months after completion of the initial therapy. The intrinsic and acquired resistance to platinum-based chemotherapy is the main reason for OvCa treatment failure [6,7].

**Figure 1:**
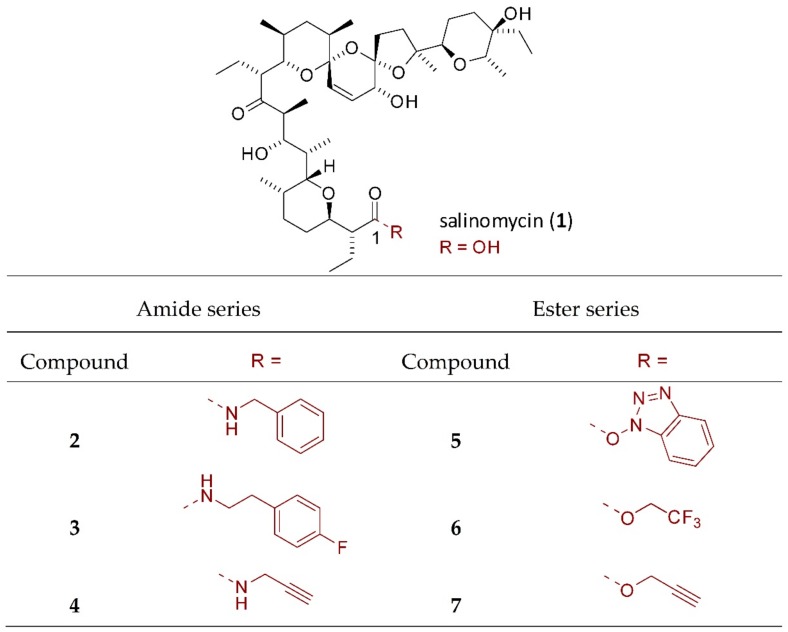
Structure of salinomycin and its derivatives studied in this work.

A few theories have been proposed to explain the development of chemoresistance in human malignancies. The most likely one is related to cancer stem cell theory [8]. According to this concept, a malignant tumor consists of two populations of cells, namely cancer stem cells (CSCs) and differentiated cells. Self-renewal, repopulation, and resistance to irradiation and cytotoxic drugs are typical of the first narrow population. Contrary to that, differentiated cancer cells are sensitive to treatment and form a bulk population of the tumor cells. CSCs, similarly to pluripotent stem cells, have active developmental signaling pathways, such as Hedgehog, Notch, and Wnt/β-catenin. The latter two seem to be particularly responsible for OvCa platinum resistance [9,10]. Unfortunately, there is no single universal marker capable of distinguishing CSCs from the differentiated cancer cells. Such a marker could help to develop a highly specific targeted therapy against CSCs, which would improve OvCa patient outcome. Therefore, the main focus of OvCa therapies should be the elimination of CSCs. One of the molecules exhibiting anticancer potential and selective properties against CSCs is salinomycin (SAL, **1**, Figure 1).

This is a monocarboxylic polyether antibiotic naturally synthesized by *Streptomyces albus* (strain no. 80614) [11]. SAL was identified in 2009 as the most active agent among 16,000 compounds tested towards breast CSCs [12]. Since then, SAL has been found effective against many other types of cancer cells and CSCs, including those displaying multidrug resistance (MDR) and has been used in a small group of patients with advanced carcinoma of the head, neck, breast, and ovary [13]. SAL acts as a sensitizer of malignant cells to radiotherapy or chemotherapy, i.e., colchicine, doxorubicin, and etoposide [14,15,16,17].

## 2. Results

### 2.1. Derivation of Cisplatin-Resistant Cell Lines

**Figure 2:**
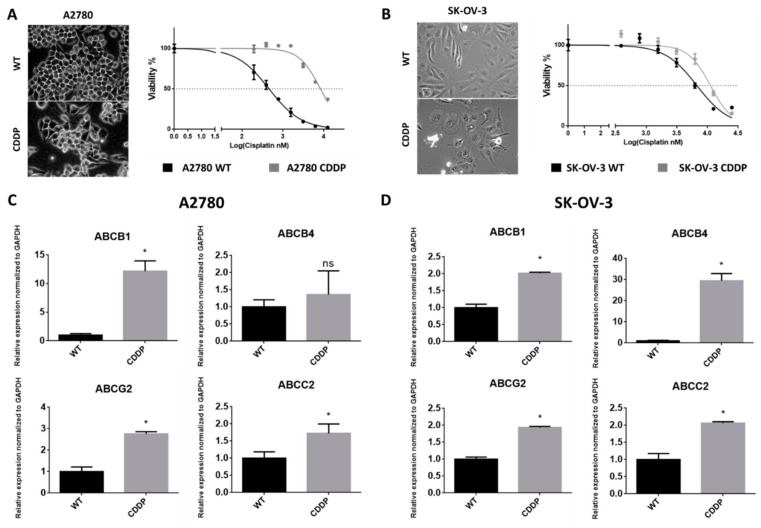
Overview of cisplatin-resistant ovarian cancer cell lines (A2780, SK-OV-3). (**A**,**B**) Morphological changes of both drug-resistant cancer sub-lines represent enlargement and slight spindle-like shape. Survival curves indicate increased IC_50_ for both resistant variants (RI = 18.08 for A2780; RI = 1.56 for SK-OV-3). The pictures were taken under 200× magnification. (**C**,**D**) RT-qPCR analysis of A2780 and SK-OV-3 revealed significantly increased expression of ABC drug transporters in derived resistant variants.

To test the usefulness of SAL and its derivatives in overcoming cisplatin-resistance, chemoresistant OvCa sub-cell lines were established. MTT and RT-qPCR followed the cell exposure to cisplatin to confirm derivation of stable phenotype of the resistant cell lines. A2780 CDDP and SK-OV-3 CDDP lines responded with morphological changes and increased IC_50_ against cisplatin as compared with their parental population (Figure 2A,B). Both resistant cell lines showed also enhanced expression of ABCB1, ABCG2, and ABCC2 versus control (Figure 2C,D). ABCB4 expression boosted significantly in SK-OV-3 CDDP cell line but only slightly in A2780 CDDP cell line.

### 2.2. In Vitro Activity of Cytotoxic Drugs, Salinomycin, and Its Derivatives Against OvCa Cells

It was clearly proven that chemical modification of SAL and other polyether ionophores may not only increase the biological activity of resulting derivatives but also reduce their general toxicity [18,19,20,21]. Furthermore, SAL with a modified C1 carboxyl group (amides or esters) transports cations by a biomimetic mechanism, while chemically unmodified SAL transports cations through biological membranes via an electroneutral mechanism [22,23]. This change in ionophoretic properties may result in better biological properties of **SAL** analogs than of those with a native structure.

We devised a library of SAL derivatives based on the most active SAL amides and esters obtained in our previous studies by a chemical modification of C1 carboxyl group, i.e., amides **2** and **3**, as well as esters **5** and **6**, respectively (Figure 1) [18,19,20]. To expand structural diversity at C1 position and to better determine the structure-activity relationship (SAR), we additionally analyzed propargyl amide **4** and propargyl ester **7** (Figure 1), as these structures had shown promising bioactivity [19].

Data gathered in Table 1 indicate that all tested compounds exhibited biological activity against malignant cells. The effect towards ovarian A2780 cell line was distinctly better than that against metastatic ovarian SK-OV-3 cell line. Briefly, the most effective was chemically unmodified SAL, the activity of which was higher against A2780 cell line and comparable against SK-OV-3 cell line than that of reference anticancer drug—cisplatin (CDDP) (Table 1). In OvCa cell lines A2780, SK-OV-3 as well as their platinum-resistant sub-lines, all semi-synthetic derivatives of SAL (both from amide and ester series) needed significantly higher IC_50_ values to induce comparable biological effects than SAL itself (Table 1). The most active SAL analog was 4-fluorophenethyl amide **3** (Figure 1) but still its activity was one order of magnitude lower than that of unmodified SAL (Table 1). As expected, cisplatin-resistant sub-lines were more resistant to CDDP than both cisplatin-sensitive variants; thus, the anticancer activity of compounds **3** and **5** (Figure 1) was higher than that exhibited by CDDP towards A2780 CDDP cell line (Table 1).

However, to the best of our knowledge, there are no reports describing the effects of a dual therapy using SAL and 5-fluorouracil (5FU) or gemcitabine (GEM) towards OvCa cells. Therefore, we decided to check if SAL shows desired results when combined with these commonly used anticancer drugs. For this purpose, we prepared 1:1 molar mixtures of SAL and 5FU/GEM (**1** + 5FU and **1** + GEM, respectively) and tested their activity towards OvCa cell lines (Table 1). Interestingly, in both cases we witnessed a strong interaction between SAL and either 5FU or GEM. IC_50_ values for **1** + 5FU and **1** + GEM against all OvCa cell lines reached a low micromolar concentration range and were significantly lower than those exhibited by individual components (**1** and 5FU) and reference anticancer drug—CDDP (Table 1). More promising (lower) IC_50_ values were only obtained for GEM (Table 1), which is recommended for treatment of recurrent OvCa.

To determine the real therapeutic potential of novel anticancer drug candidates, it is necessary to check their effects (selectivity) towards normal cells. Judging by IC_50_ values, the tests performed in normal diploid human MRC-5 cell line suggested that all SAL derivatives were significantly less toxic towards non-malignant human cells than the chemically unmodified SAL (Table 1).

### 2.3. Salinomycin and Its Derivatives Overcome Cisplatin Resistance and are More Selective Against Cisplatin-Resistant OvCa Cells

**Figure 3:**
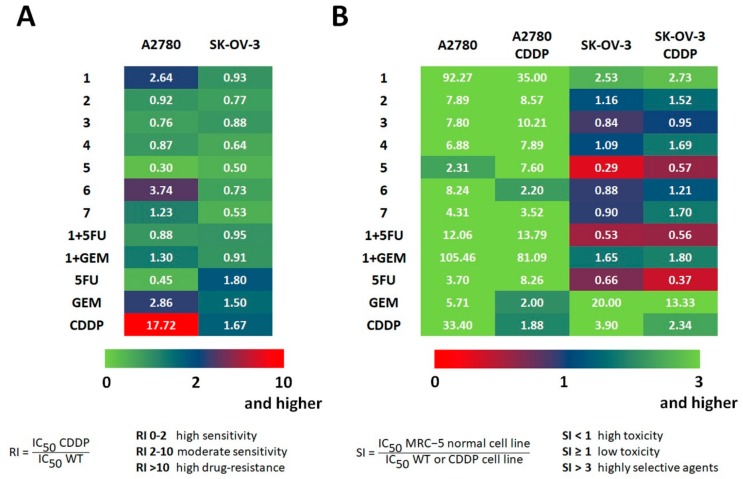
Calculated values of (**A**) the resistance indexes (RI), and (**B**) selectivity indexes (SI) of the tested compounds.

To assess the effectiveness of the studied drugs in overcoming acquired cisplatin resistance, we calculated the resistance index (RI), based on the IC_50_ of derived cell lines (Figure 3A). The RI indicates how many more times a resistant sub-line (either A2780 CDDP or SK-OV-3 CDDP) is chemoresistant as compared with its parental cell line (either A2780 or SK-OV-3, respectively). An RI between 0 and 2 indicates that the cells are sensitive to the tested compound. An RI in the range from 2 to 10 means that the cells show moderate sensitivity to a drug. An RI above 10 indicates strong drug resistance [24]. None of the cancer cell lines developed strong resistance to the tested agents under the combinatory treatment of SAL with either 5FU or GEM (Figure 3A). Contrary to GEM, both **1** + 5FU and **1** + GEM co-treatments were capable of efficiently overcoming the drug resistance of OvCa cells, as manifested by considerably lower values of RI (Figure 3A).

Deeper analysis of RI parameters revealed that cancer cell line A2780, in opposition to SK-OV-3, developed some resistance to SAL (Figure 3A). This finding may indicate possible treatment failure on SAL clinical application. On the other hand, almost no SAL derivatives (except for **6** and **7**) developed even mild resistance during our experiments in both OvCa cell lines (Figure 3A). Generally, A2780 and SK-OV-3 cell lines turned out more sensitive to amide analogs of SAL (compounds **2**–**4**) than the corresponding ester derivatives (compounds **6**–**7**). The only exception to this rule was ester **5** (RI = 0.30 and RI = 0.50 for A2780 and SK-OV-3 cell line, respectively) (Figure 3A).

Further, to establish therapeutic potential of the tested anticancer agents, we used the difference in the antiproliferative activity towards the OvCa cell lines and the corresponding normal cell line to calculate the values of the selectivity index (SI) (Figure 3B). The SI is an important pharmaceutical parameter that facilitates the estimation of possible future clinical development; higher values of SI indicate greater anticancer specificity, and the compounds displaying an SI above 3.0 are considered highly selective agents [21,25]. Therefore, our study clearly proved that normal cells were less sensitive to SAL used alone or its combinations with other cytotoxic agents (5FU or GEM) than to amide and ester derivatives of SAL (Figure 3B).

Selectivity of SAL as well as its amide and ester analogs was very promising, especially in non-resistant and resistant to cisplatin A2780 cell lines (Figure 3B). However, the highest values of SI were noted for SAL and its 1:1 molar mixture with GEM (**1** + GEM, Figure 3B), and this effect decreased with time of exposure to the anticancer agents. On the other hand, SAL turned out more selective against SK-OV-3 cell line and its platinum-resistant variant than all analyzed amide and ester derivatives (Figure 3B). A combinatory treatment of OvCa cells involving SAL and GEM (**1** + GEM, Figure 3B) yielded very high SI values. The combination of the cytotoxic agents (regardless of their mechanism of anticancer action), either 5FU or GEM, with SAL was more effective than SAL or its derivatives used alone (Table 1 and Figure 3).

As OvCa cells seemed highly sensitive to the action of both **1** + 5FU and **1** + GEM (Table 1 and Figure 3), we decided to determine the exact effect (addictive, synergistic, or antagonistic) of the specific compound combinations. Using the 1:1 molar mixtures of SAL and 5FU or GEM, we performed standard viability assays and subjected the results to the analysis and determination of values of the combination indexes (CI) (Table 2). As previously described for a pair of drugs [26,27], CI > 1.3 indicates antagonism, CI = 1.1–1.3 indicates moderate antagonism, CI = 0.9–1.1 indicates additive effect, CI = 0.8–0.9 indicates slight synergism, CI = 0.6–0.8 indicates moderate synergism, CI = 0.4–0.6 indicates synergism, and CI = 0.2–0.4 indicates strong synergism. Interestingly, the results presented in Table 2 indicated that SAL in combination with 5FU acted synergistically (CI = 0.56) in A2780 CDDP cell line, whereas in SK-OV-3 and SK-OV-3 CDDP cell lines, they only showed an additive effect (CI = 1.09 and CI = 1.02, respectively).

**Figure 4:**
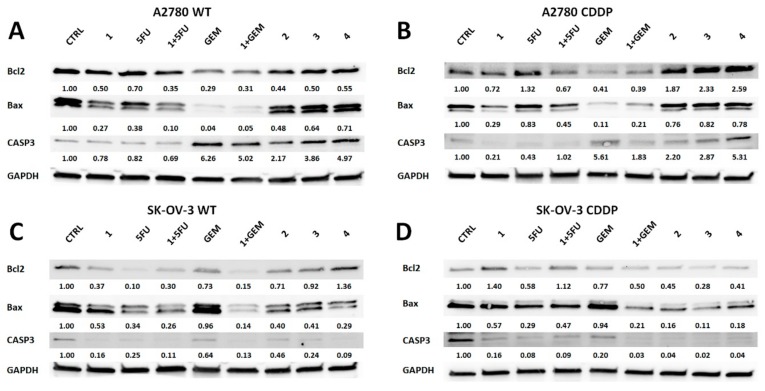
Western blot analysis of OvCa cell lines exposed to IC_50_ values of selected compounds for 72 h (**A**–**D**). The expression of anti-apoptotic (Bcl2) and apoptotic proteins (Bax and CASP3) was evaluated. The numbers describe the quantified level of band intensity normalized to expression of reference protein GAPDH and the control population.

After a primary screening of the compounds, SAL and its amides indicated in both OvCa cell lines variants the most promising response. To confirm their effect, Western blot analysis for B-cell lymphoma 2 (Bcl2), Bcl2 associated X protein (Bax), and caspase-3 (CASP3) was performed (Figure 4).

In A2780 cells, the Bcl2 decreased in all groups in comparison with the control (Figure 4A). The lowest expression was observed in the GEM and 1 + GEM variant, but their effect was similar. The proapoptotic protein Bax also represented similar trends of expression as Bcl2. Interestingly, full form of caspase-3 (CASP3) was decreased in cells treated with SAL, 5FU, and their combination, but GEM and its combination with SAL, as well as amide derivatives of SAL, caused the enhanced expression of that protein. The antagonistic effect of combined SAL with 5FU or GEM was observed by decreasing expression of CASP3. In case of the A2780 CDDP variant, Bcl2 expression in cells treated with SAL, 1 + 5FU, GEM, and 1 + GEM was decreased (Figure 4B). Surprisingly, the SAL amides (**3**–**5**) caused increased Bcl2 expression. It is worth mentioning that a combination of 5FU with SAL caused downregulation of Bcl2. In all tested compounds the expression of Bax was inhibited in comparison with the control. Among them, a combination of SAL with 5FU caused decreased expression of Bax compared to 5FU alone. A similar effect onto Bax expression was observed in the GEM and its combination with SAL. In A2780 CDDP cell line, CASP3 expression in SAL and 5FU was decreased, and their combination caused a similar effect in comparison to control. However, the expression of that protein in GEM, 1 + GEM, 3, 4, and 5 variants was enhanced in comparison with the control. The combination of SAL with 5FU caused the enhanced expression of the full form of CASP3, which confirmed synergistic action of these compounds. On the other hand, the antagonistic effect was observed in GEM and its combination with SAL, where the cytotoxic effect was enhanced in only GEM-treated cells.

In SK-OV-3 cell line, Bcl2 expression was decreased in all studied compounds, except amide **4**, compared to control (Figure 4C). Among them, SAL, 5FU, and **1**+GEM showed the lowest expression. All tested compounds indicated decreased expression of Bax in comparison with control. The combination of SAL and 5FU did not cause the upregulation of these proteins exciding the level of compounds tested alone. The GEM indicated the highest expression level of Bax among tested molecules. Amide derivatives of SAL did not indicate increased expression of Bax in comparison with SAL. The similar tendencies of CASP3 expression were observed, excluding compound **2** and **3**, which exhibited its higher expression in comparison with SAL. These observations confirmed the antagonistic effect in studied combinations of antineoplastic agents with SAL. In the case of SK-OV-3 CDDP cell line exposed to distinct molecules, the expression of Bcl2 only in SAL and SAL combined with 5FU was upregulated in comparison with the control (Figure 4D). Only the combination of SAL with GEM caused enhanced downregulation of Bcl-2 compared to exposition to them alone. Bax expression in those cells was decreased in comparison with the control. Among tested variants, 1 + 5FU exceeded the level of its expression compared to 5FU, but not to SAL alone. Again, GEM used separately induced its highest expression among all exposed compounds. The amide derivatives of SAL indicated lower expression of Bax in comparison with SAL. The presence of Bax downregulation in cells exposed to a combination of antineoplastics with SAL confirmed their antagonism. The expression of CASP3 and the action of compounds co-cultured with SK-OV-3 CDDP cells was similar to that of Bax. These results confirmed earlier estimated effects of combined SAL with 5FU or GEM in studied cell lines.

## 3. Discussion

One of the major problems in ovarian cancer (OvCa) treatment is development of chemoresistant residual cancer cells. Cancer stem cell (CSC)-targeted therapies should thus be developed [5]. One of the promising compounds in this respect is salinomycin (SAL). Its short-lasting side effects and low solubility in aqueous solutions may be avoided by some chemical modifications. These modifications provide new SAL molecules with improved stability and unaffected selective properties against CSCs, particularly effective in overcoming chemoresistant forms of the disease [28,29]. However, the synthesis of selective SAL derivatives is complicated due to the presence of multiple functional groups and a sensitive tricyclic 6-6-5 bis-spiroketal ring system in SAL structure [20,29,30].

In this study, we derived cisplatin-resistant cell lines A2780 CDDP and SK-OV-3 CDDP and evaluated the antiproliferative activity of selected amides and esters of SAL, as well as SAL used alone or in combination with other commonly used cytostatic agents, such as 5-fluorouracil (5FU) and gemcitabine (GEM), against these OvCa cells. To confirm the observed effect, Western blot analysis of proteins related to apoptosis (Bcl2, Bax and CASP3) was also performed.

We obtained resistant variants of cancer cell lines, as confirmed by increased IC_50_ against cisplatin estimated by MTT assay. These variants also showed elevated expression of genes related to drug transporters. ABCB1, ABCB4, ABCG2, and ABCC2 are well-described markers of drug resistance development in many cancers [31,32,33,34,35]. These proteins are characteristic of side populations of cells that represent stem-cell-like features, and in some cancers, they are recognized as markers of CSCs. They are responsible for active efflux of many anticancer agents causing treatment failure [35,36,37].

Our experiments provided interesting data regarding the activity of SAL and its derivatives against platinum resistance developed in OvCa cell lines in vitro. Creation of platinum-resistant OvCa cell line (SK-OV-3) did not induce the resistance of cancer cells to the action of SAL and its derivatives. This indicated that different molecular mechanisms are responsible for either platinum or SAL resistance, and additionally SAL could be identified as an effective agent in overcoming the platinum resistance of OvCa cells. However, A2780 CDDP variant exhibited moderate sensitivity to SAL and its 2,2,2-trifluoroethyl ester derivative (compound **6**, Figure 1). This phenomenon could be related to the increased expression of ABCB1 and ABCG2 in resistant variants of A2780 cancer cell line. A study by the Boesch group revealed that in OvCa cell lines (A2780, IGROV1), selected ABCB1 and ABCG2 positive cells exhibited protective mechanisms against ionophore antibiotics, including SAL [38]. Contrary to that, a study in 2010 demonstrated that SAL treatments restored doxorubicin sensitivity in human doxorubicin-resistant epithelial OvCa cell line (A2780/ADR) exposed to doxorubicin either alone or in combination with SAL by inhibition of ABCB1 functionality [39]. Disparate results of these studies might be related to distinct co-activation of a group of genes in the same chromosomal region, where ABCB1 activation occurs by various cytotoxic agents [31].

Our experiments did not confirm the high anticancer activity of SAL derivatives reported recently in primary acute lymphoblastic leukemia (ALL) cells [20,40], which may be caused by a different type of malignancies and their adverse biology; ALL represents a hematological malignancy, while OvCa represents a malignant solid tumor. Until now, there have been only limited data showing the effects of SAL and its derivatives on OvCa cells. The recent data indicated that SAL itself affects a wide spectrum of mechanisms against OvCa biology, such as inhibition of the epithelial-mesenchymal transition (EMT) process (responsible for the development of metastatic disease), eradication of the CSC population (CD44^+^CD117^+^), and inhibition of the NF-ĸB signaling pathway (upregulation of proteins related to that pathway correspond with poor clinical prognosis in OvCa) [41,42,43,44]. Our study, for the first time, demonstrates that the combination of SAL with cytostatic agents (5FU and GEM) is more effective than SAL used alone or its amide and ester derivatives. 5FU is not commonly used in OvCa treatment—a few clinical trials revealed no significant improvement in clinical outcomes in advanced OvCa patients receiving 5FU combined with cisplatin or leucovorin [45,46,47]. Of note is that SAL acted synergistically with 5FU towards drug-resistant A2780 OvCa cell line. A similar effect was presented in studies concerning colorectal and hepatocellular carcinoma, which indicated the neutral or synergistic effect of the combination of SAL and 5FU [48,49]. In case of GEM, which is mostly used as a secondary line of chemotherapy after the development of resistant disease in OvCa, we did not observe the synergistic effect of combined SAL and GEM in both OvCa cell lines, as was found over their action in pancreatic carcinoma cell lines [50,51]. We think that one of the causes was their high molar ratio 1:1 used in our study versus 5 μM concentration of SAL and 5 µg·mL^−1^ applied in the pancreatic cancer cell lines [51]. The differences between the observed effects of these two nucleoside analogs in combination with SAL might be related to a disturbance of their cellular uptake by an ion imbalance caused by SAL. The concentrative nucleoside transporters (CNT) and equilibrative nucleoside transporters (ENT) are mostly engaged in the transport of GEM, while for 5FU, only ENT proteins are involved [52,53,54,55]. The mechanism of nucleoside transport by CNT proteins is Na^+^-dependent, whereas in the case of ENT, they are mediated by facilitated diffusion [53,54]. SAL is responsible for the transport of potassium and sodium cations, which could lead to disturbance of the GEM uptake through CNT transporters (indicating high affinity to GEM), decreasing its activity against cancer cells [29,54,55].

The effect of combination of SAL with GEM or 5FU was confirmed by Western blot analysis of proteins related to apoptosis (Bcl2, Bax, and CASP3). Besides the same IC_50_ effect at distinct concentrations of studied compounds, SAL amide derivatives (3–5) caused enhanced expression of CASP3 in A2780 and A2780 CDDP cell lines, which could suggest different or more effective induction of apoptosis by these molecules. We did not observe a similar effect in SK-OV-3 cells, which could be related to intrinsic resistance mechanisms due to their metastatic origin. However, there is a lack of detailed mechanistic studies explaining the differences between SAL and amide derivatives against OvCa cell biology; thus, their further detailed characterization of action should be performed. On the other hand, for SAL used alone, we obtained similar findings as the Parajuli group, where the A2780 CDDP variant was tested, and a decrease of the Bcl2, Bax, and CASP3 was noted at similar doses that we used in our study; however, in their study, only after 1 µM of SAL, caspases were significantly increased [44,56]. They also indicated that the cause of apoptosis is related to activation of death receptor 5 (DR5) pathway, which was observed even at low doses of SAL (0.5 µM) [56]. In contrast, the Kaplan group observed induction of apoptosis by upregulation of CASP3 in OVCAR3 cell line even at 0.1 µM of SAL [57].

In summary, all findings mentioned above corresponded well with our results. Potent anticancer activity of SAL, lack of SAL resistance in platinum-resistant OvCa cell lines, and reversible SAL resistance imply the possible application of SAL in overcoming either primary or acquired platinum resistance (in platinum-resistant cell lines/patients). Further analyses of the SAL treatment used alone or in combination with other anticancer drugs, such as 5FU and GEM, will require identification of the most effective combination of SAL and the cytotoxic agent, and the mode of their administration (synchronous or sequential). Our results may contribute to the development of anticancer therapy based on SAL, which may give hope for heavily treated OvCa patients. Further studies should focus on discovering the mechanisms of action for SAL and its derivatives.

## 4. Materials and Methods

### 4.1. Chemical Part

#### 4.1.1. Isolation of Salinomycin

Salinomycin sodium salt was isolated from commercially available veterinary premix SACOX^®^ following acidic extraction, using the previously described procedure [18,19]. Briefly, isolated sodium salt of salinomycin was dissolved in CH_2_Cl_2_ and stirred vigorously with a layer of aqueous sulfuric acid (pH = 1.0). The organic layer containing salinomycin (SAL, **1**, Figure 1) was washed with distilled water. Then, CH_2_Cl_2_ was evaporated under reduced pressure to dryness giving SAL as clear oil. After three cycles of evaporation with *n*-pentane, this oil was transformed into white amorphous solid. Spectroscopic data for SAL were closely matched previously published data [22].

#### 4.1.2. Synthesis of Salinomycin Derivatives

All SAL amides and esters (compounds **2**–**7**, Figure 1) obtained by a chemical modification of the C1 carboxyl group were prepared according to the procedures we described previously [18,19]. Spectroscopic data of all the compounds matched those found in the reference literature [18,19].

### 4.2. Biological Part

#### 4.2.1. Cell Culture and Derivation of Cisplatin-Resistant Cell Lines

In this study, OvCa cell lines A2780 and SK-OV-3 (ATCC, Manassas, VA, USA) and human fetal lung fibroblasts cell line (MRC-5 pd19; ECACC, Salisbury, United Kingdom) were used to evaluate the antiproliferative activity of the tested compounds. OvCa cell lines were cultivated in RPMI 1640 containing 25 mM HEPES and 5 mM l-glutamine with 10% fetal bovine serum (FBS) (all provided from Biowest, Nuaillé, France) and 1% penicillin-streptomycin (Merck KGaA, Darmstadt, Germany). MRC-5 pd19 was cultured in DMEM supplemented with 10% FBS, 2 mM l-glutamine (all provided from Biowest, Nuaillé, France), 1% penicillin-streptomycin and 1% non-essential amino acids (NEAA) (both provided from Merck KGa, Darmstadt, Germany). To generate the cisplatin-resistant cell lines (A2780 CDDP; SK-OV-3 CDDP), increasing doses of cisplatin (CDDP) (Teva Pharmaceutical Industries Ltd. Petach Tikwa, Israel) were added to the culture medium, starting from the concentration of 100 ng mL^−1^. Then, the cells were exposed to CDDP (3 cycles of 3 days each). After that, the cell culture medium was replaced with the fresh one without drugs for the next 3 days or one week until the cells recovered. After the 3 cycles, the dose of cisplatin was doubled until the concentration of 1000 ng·mL^−1^ was achieved. Then, to maintain a resistant phenotype, 1000 ng·mL^−1^ of **CDDP** was added once per 2 weeks for 3 days.

#### 4.2.2. Isolation of RNA and RT-qPCR

The cells (2.5 × 10^5^) were washed twice in Dulbecco’s phosphate buffered saline (DPBS, Biowest, Nuaillé, France) and suspended in TRI reagent (Sigma-Aldrich, St. Louis, MO, USA). Next, RNA was isolated using Direct-zol RNA MiniPrep (Zymoresearch, Irvine, CA, USA) according to the manufacturer’s instructions. Then 1 µg of RNA was collected to synthesize cDNA using iScript kit (BioRAD, Hercules, CA, USA). The cDNA was diluted 20 times, and 2.5 µL was added to the reaction mix composed of FastStart Essential DNA Probes Mix and specific probes (both provided by Roche Molecular Systems, Inc, Basel, Switzerland). The RT-qPCR reaction was performed as previously described [58]. The tested genes related to drug resistance included: ATP-binding cassette subfamily B member 1 (ABCB1), ATP-binding cassette subfamily B member 4 (ABCB4), ATP-binding cassette subfamily G member 2 (ABCG2), and ATP-binding cassette subfamily C member 2 (ABCC2). Relative gene expression level was determined using reference gene glyceraldehyde 3-phosphate dehydrogenase (GAPDH). The primers and molecular probes used in this study are listed in Table S1 (Supplementary Material).

#### 4.2.3. Cell Viability Assay

For the evaluation of cell proliferation inhibition, we used the MTT (3-(4,5-dimethylthiazol-2-yl)-2,5-diphenyltetrazolium bromide) assay as previously described [59]. Briefly, A2780 WT and CDDP (2000 cells/well), SK-OV-3 WT and CDDP (800 cells/well), and MRC-5 pd19 (3000 cells/well) were seeded onto a 96-well plate for overnight until the cells attached. Next, the cells were exposed to the estimated concentrations of SAL, its derivatives, and anticancer drugs for 72 h (Table S2, Supplementary Material). Then the cell culture medium was replaced with the fresh one containing 0.5 mg·mL^−1^ of MTT (Affymetrix, Santa Clara, MA, USA) and left for 2 h at 37 °C. After that, the medium was replaced with DMSO (VWR, Darmstadt, Germany) and left at 37 °C for 10 min until the crystals were dissolved. The measurement was performed with a plate reader, Multiskan FC (Thermofisher, San Jose CA, USA) at 570 and 690 nm. Then, IC_50_ and mean 95% CI were determined using GraphPad Prism 6 (Graph Pad Software, San Diego, CA, USA). The values of resistance index (RI), selectivity index (SI), and combination index (CI) were calculated as described previously [20,60].

#### 4.2.4. Western Blot Analysis

After 72 h of exposure to IC_50_ concentration of selected compounds, the OvCa cell lines were lysed using RIPA lysing buffer (Sigma Aldrich, St. Louis, MO, USA). For analysis, 10 µg of protein was used, calculated using Pierce™ BCA Protein Assay Kit (Thermofisher, San Jose, CA, USA) according to the manufacturer’s instructions. The procedure was performed as previously described [52]. Briefly, after electrophoresis, proteins were transferred onto polyvinylidene difluoride (PVDF) membrane. After 2 h of blocking with non-fat milk (Sigma Aldrich, St. Louis, MO, USA), the membrane was incubated overnight at 4 °C with primary antibodies: anti-Bcl-2 (dilution 1:500; sc-509, Santa Cruz, Dallas, TX, USA), anti-Bax (dilution 1:500; sc-7480, Santa Cruz, Dallas, TX, USA), anti-CASP3 (full-form)(dilution 1:1000; no. ab49822, Abcam, Cambridge, UK), and reference protein GAPDH (dilution 1:500; no. sc-47724, Santa Cruz, Dallas, TX, USA). On the next day, after washes, the membranes were incubated with appropriate secondary antibodies conjugated with horseradish peroxidase (HRP) (dilution 1:1000; no. 7076 and 7074, Cell Signaling Technology, Leiden, Netherlands). Then the protein bands were visualized using WesternBright™ Quantum kit (Advansta, San Jose, CA, USA) and documented using the ChemiDoc Touch Imaging System (Bio-Rad Laboratories Ltd., Hercules, CA, USA). The intensity was measured using Image Lab Software (ver 6.0.1, Bio-Rad Laboratories Ltd., CA, USA). All buffers and equipment used during Western blot analysis were provided from Bio-Rad Laboratories Ltd., CA, USA.

#### 4.2.5. Statistical Analysis

The statistical analysis of genes expression (Student’s *t*-test) was performed using GraphPadPrism 6 package (Graph Pad Software, San Diego, CA, USA). The data were deemed significant at *p* < 0.05. All experiments were repeated at least three times.

## Figures and Tables

**Table 1:** The IC_50_ values estimated for ovarian cancer cell lines (A2780, SK-OV-3, both drug-sensitive and drug-resistant variants) and normal diploid human MRC-5 cell line after 72 h exposure to salinomycin (**SAL**, **1**), its 1:1 molar mixtures with cytotoxic drugs (5-fluorouracil **5FU**, gemcitabine **GEM**), and salinomycin amides and esters (analogs **2–7**).

Compound		A2780		A2780 CDDP		SK-OV-3		SK-OV-3 CDDP		MRC-5	
		IC_50_ (µM)	CI 95%	IC_50_ (µM)	CI 95%	IC_50_ (µM)	CI 95%	IC_50_ (µM)	CI 95%	IC_50_ (µM)	CI 95%
**SAL**	**1**	0.11	0.08‒0.13	0.29	0.27‒0.29	4.01	3.13‒5.11	3.72	3.24‒4.25	10.15	5.14–20.01
salinomycin amides	**2**	27.05	24.25‒30.18	24.90	19.98‒31.04	183.45	156.43‒215.24	140.60	119.40‒165.71	213.33	186.19–244.52
	**3**	8.49	6.74‒10.68	6.48	4.71‒8.89	79.08	71.25‒87.76	69.40	61.88‒77.84	66.19	57.31–83.54
	**4**	14.38	11.99‒17.23	12.54	9.58‒16.40	91.17	81.42‒102.07	58.53	53.22‒64.37	98.96	88.13–111.13
salinomycin esters	**5**	25.50	20.44‒31.79	7.74	6.04‒9.94	205.88	188.71‒224.65	103.41	82.63‒129.38	58.81	47.05–73.53
	**6**	13.41	10.58‒17.01	50.16	39.26‒64.08	125.33	117.36‒133.85	91.58	83.78‒100.11	110.46	98.87–123.41
	**7**	37.31	27.95‒49.56	45.74	35.49‒57.05	179.72	146.77‒219.90	94.56	85.65‒104.41	160.96	96.41–268.69
1:1 molar mixtures	**1 + 5FU**	0.16	0.14‒0.19	0.14	0.14‒0.15	3.65	2.96‒4.49	3.45	2.91‒4.11	1.93	0.83–4.49
	**1 + GEM**	0.018	0.01‒0.03	0.024	0.01‒0.04	1.17	1.07‒1.29	1.07	1.07‒1.29	1.93	1.09–3.43
reference anticancer drugs	**5FU**	3.62	2.15‒6.00	1.62	1.23‒2.08	20.23	8.85‒46.23	36.38	16.85‒78.46	13.38	7.23–24.75
	**GEM**	0.007	0.006‒0.007	0.02	0.01‒0.02	0.002	0.00007‒0.01	0.003	0.00003‒0.02	0.04	0.01–0.012
	**CDDP**	0.47	0.40‒0.50	8.33	7.40‒9.37	4.03	3.48‒4.61	6.72	5.96‒7.56	15.7	12.9 –19.27

**Table 2:** Calculated combination index (CI) values of simultaneously delivered salinomycin (**SAL**, **1**) and cytotoxic drugs (5-fluorouracil **5FU**, gemcitabine **GEM**) in the 1:1 molar mixtures.

Combination of Compounds	A2780	A2780 CDDP	SK-OV-3	SK-OV-3 CDDP
1+5FU	1.57	0.56	1.09	1.02
1+GEM	2.86	1.60	691	360

## Supplementary Materials

The following are available online, Table S1: List of primers used in this study, Table S2: Concentration of drugs, dilution range, and serial dilution factors used in this study.

## Abbreviations

5FU	5-fluorouracil
ABCB1	ATP-binding cassette subfamily B member 1
ABCB4	ATP-binding cassette subfamily B member 4
ABCC2	ATP-binding cassette subfamily C member 2
ABCG2	ATP-binding cassette subfamily G member 2
ALL	Acute lymphoblastic leukemia
Bax	Bcl2 associated X protein
Bcl-2	B-cell lymphoma 2
CASP3	Caspase 3
CDDP	Cisplatin
CI	Combination index
CNT	Concentrative nucleoside transporters
CSCs	Cancer stem cells
DMEM	Dulbecco modified eagle medium
EMT	Epithelial-mesenchymal transition
ENT	Equilibrative nucleoside transporters
FBS	Fetal bovine serum
GAPDH	Glyceraldehyde 3-phosphate dehydrogenase
GEM	Gemcitabine
HRP	Horseradish peroxidase
MDR	Multidrug resistance
MTT	3-(4,5-dimethylthiazol-2-yl)-2,5-diphenyltetrazolium bromide
NEAA	Non-essential amino acids
OvCa	Ovarian cancer
P-gp	P-glycoprotein
PVDF	Polyvinylidene difluoride
RI	Resistance indexes
RT-qPCR	Reverse transcriptase quantitative polymerase chain reaction
SAR	Structure-activity relationship
SI	Selectivity indexes
