# Supplementary material for: Overcoming Resistance to Platinum-Based Drugs in Ovarian Cancer by Salinomycin and Its Derivatives—An In Vitro Study
Source: Molecules. 2020 Jan 26;25(3):537. doi: 10.3390/molecules25030537 (PMC7037477; doi:10.3390/molecules25030537)
Supplement: Supplementary file 1 [file molecules-25-00537-s001.pdf]

Table S1. The list of primers used in this study.

	Forward sequence (5′-3′)	Reverse sequence (5′-3′)	Amplicon	No. of Molecular probe
**ABCB1**	AGCCCTTGGAATTATTTCTTTTATTA	ATGTATCGGAGCCGCTTG	93 bp	78#
**ABCB4**	AACCTCTGATTGACAGCTACAGTG	GGGTGGGATAGTTGAACACG	96 bp	41#
**ABCG2**	GCAACAGGAAACAATCCTTGTAA	AAGGCCACGTGATTCTTCC	110 bp	45#
**ABCC2**	TGCTGGGAGAAATGGAAAAT	CAGGACTGCTGTGGGACATA	76 bp	9#
**GAPDH**	TCCACTGGCGTCTTCACC	GGCAGAGATGATGACCCTTTT	78 bp	45#

Table S2. The concentration of drugs, dilution range and serial dilution factors used in present study.

		Dilutions range (μg mL^{-1})					
	Stock (mg mL^{-1})	A2780	A2780 CDDP	SK-OV-3	SK-OV-3 CDDP	MRC-5 pd19	Serial Dilution factor
**1**	25	10–0.156	10–0.156	100–1.56	100–1.56	100–1.56	2x
**1+5FU**	25	10–0.156	10–0.156	100–1.56	100–1.56	100–1.56	2x
**1+GEM**	25	10–0.156	10–0.156	100–1.56	100–1.56	100–1.56	2x
**2**	25	100–1.56	100–1.56	300–4.68	400–6.25	400–6.25	2x
**3**	20	100–1.56	100–1.56	300–4.68	300–4.68	300–4.68	2x
**4**	20	100–1.56	100–1.56	300–4.68	400–6.25	400–6.25	2x
**5**	25	100–1.56	100–1.56	300–4.68	400–6.25	400–6.25	2x
**6**	25	100–1.56	100–1.56	300–4.68	400–6.25	400–6.25	2x
**7**	25	100–1.56	100–1.56	300–4.68	400–6.25	400–6.25	2x
**CDDP**	1	15–0.23	15–0.23	15–0.23	15–0.23	30–0.47	2x
**5FU**	50	100–0.0001	100–0.0001	200–0.0002	200–0.0002	200–0.0002	10x
**GEM**	100	100–0.0001	100–0.0001	100–0.0001	100–0.0001	10000–0.01	10x
